# Anémie de fanconi au CHU Hassan II Fès: à propos de 6 observations

**DOI:** 10.11604/pamj.2017.28.286.4372

**Published:** 2017-12-04

**Authors:** Laila Bouguenouch, Imane Samri, Meryem Abbassi, Hasna Hamdaoui, Ihssane El Otmani, Hanane Sayel, Said Trhanint, Sara Benmiloud, Moncif Amrani, Sanae Bennis, Karim Ouldim, Mustapha Hida

**Affiliations:** 1 Unité de Génétique Médicale et d'Oncogénétique, Laboratoire Centrale d'Analyses Médicales, CHU Hassan II, Fès, Maroc; 2 Faculté de Médecine et de Pharmacie de Fès, Maroc; 3 Service de Pédiatrie CHU Hassan II Fès, Maroc; 4 Laboratoire d’'Hématologie, Laboratoire Centrale d'Analyses Médicales, CHU Hassan II, Fès, Maroc

**Keywords:** Fanconi anaemia, mitomycin C, chromosome breaks, pancytopenia, Anémie de fanconi, mitomycine C, cassures chromosomiques, pancytopénie

## Abstract

L'anémie de Fanconi est une maladie récessive associée à une instabilité chromosomique, elle est marquée par une hétérogénéité phénotypique qui inclut une insuffisance médullaire, un syndrome malformatif variable, une prédisposition à développer des leucémies aiguës myéloïdes (LAM) et une hypersensibilité cellulaire aux agents pontant l'ADN. Le diagnostic est basé sur l'augmentation anormale du taux de cassures chromosomiques spontanées mais surtout, et de manière spécifique, sur une augmentation nette de ces cassures chromosomiques en présence d'agents alkylants bifonctionnels, ce qui est le cas pour nos six patients. Le conseil génétique rejoint celui des maladies autosomiques récessives. Nous rapportons nos premières observations au CHU Hassan II Fès, confirmées par la mise en évidence d'une grande instabilité chromosomique après culture sous *Mitomycine C* en comparaison avec un témoin normal. Le but de cet article est la mise à jour de nos connaissances sur la génétique de l'Anémie de Fanconi et à travers ces six observations nous illustrons le rôle de la cytogénétique dans le diagnostic et le conseil génétique pour une meilleure prise en charge aussi bien des enfants atteints que de leurs familles.

## Abstract (en)

Fanconi anemia is a recessive disorder associated with chromosomal instability. It is marked by phenotypical heterogeneity which includes medullary deficiency, a variable malformation syndrome, a predisposition to develop acute leukaemias myéloïdes (ALM) and a cellular over-sensitiveness with the agents bridging the ADN. The diagnosis is based on the abnormal increase in the rate of spontaneous breaks chromosomal but especially and in a specific way, on a clear increase in these chromosomal breaks in the presence of bifunctional alkylating agents, which is the case in our six patients. Genetic counseling is that available for autosomal recessive diseases. We report our initial observations conducted at the University Hospital (CHU) Hassan II of Fez confirmed by the detection of a large chromosomal instability after culture with Mitomycin C compared to a normal control group. The purpose of this study was to update our knowledge of Fanconi anemia genes and to highlight the role of cytogenetics in its diagnosis and the genetic counseling for better management of affected children and their families.

## Introduction

L'anémie de Fanconi est une maladie rare, à transmission récessive qui entre dans le cadre des syndromes d'instabilité chromosomique. Elle est caractérisée par une aplasie médullaire d'apparition progressive, des malformations congénitales diverses et surtout un risque accru de cancers hématologiques, en particulier les leucémies aiguës myéloïdes et de certaines tumeurs solides [1, 2]. Le phénotype de l'anémie de Fanconi est très hétérogène, certains patients ont des anomalies morphologiques importantes et une pancytopénie de début précoce, d'autres, au contraire, n'ont que peu ou pas d'anomalies morphologiques et ne développant que tardivement une pancytopénie [3]. Les premiers signes de pancytopénie surviennent en général entre trois et dix ans. En dehors des troubles morphométriques (petite taille, microcéphalie), les anomalies le plus souvent rencontrées sont des anomalies de la pigmentation cutanée, des malformations squelettiques affectant surtout les membres supérieurs et des anomalies rénales [4]. L'association à certaines endocrinopathies est relativement fréquente [5]. Le taux de cassures chromosomiques spontanées est généralement augmenté chez les patients atteints d'anémie de Fanconi mais le diagnostic de certitude se base sur l'hypersensibilité des cellules des patients atteints d'anémie de Fanconi vis-à-vis des agents alkylants bifonctionnels. Sur le plan cellulaire, 15 gènes ont été mis en évidence dont la mutation peut être responsable d'AF. Ces gènes sont impliqués dans une voie de réparation des lésions acquises de l'ADN, la voie FANC/ BRCA. La greffe médullaire représente jusqu'ici la seule possibilité pour les patients atteints d'anémie de Fanconi de guérir l'aplasie médullaire et de prévenir la leucémie [6]. A travers nos six observations colligées diagnostiquées et suivies dans notre institution, nous mettrons à jour les dernières actualités scientifiques et en valeur l'intérêt d'une approche multidisciplinaire.

## Méthodes

**Figure 1:**
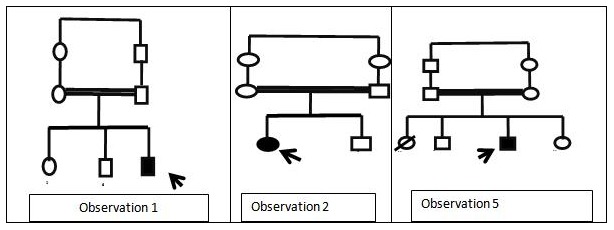
Arbres généalogiques des patients

Il s'agit d'une étude descriptive rétrospective au sein de l'unité de génétique médicale et d'oncogénétique du CHU Hassan II Fès. Notre population d'étude était constituée de 6 cas d'Anémie de Fanconi, enregistrés entre 2010 et 2013. Les données ont été recueillies à travers les dossiers médicaux des malades. Il s'agissait des arbres généalogiques (Figure 1), caractéristiques sociodémographiques, données cliniques, para cliniques et thérapeutiques.

### Observations cliniques

**Figure 2:**
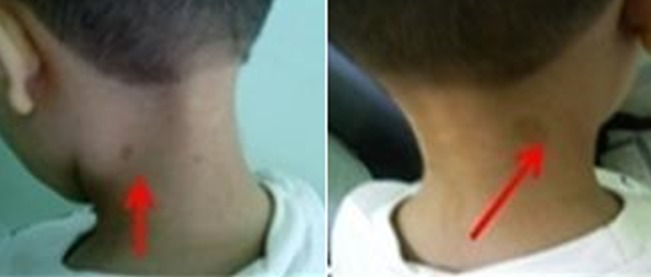
Taches café au lait, observation 1

**Observation 1**: Il s'agit de l'enfant JA âgé de 12 ans, issu d'un mariage consanguin de premier degré. L'examen clinique trouve un retard staturo-pondéral (poids-2DS, taille-2DS), une dysmorphie faciale faite d'un visage triangulaire avec des traits fins et micrognatisme, des taches café au lait au niveau de la nuque et une hyperpigmentation cutané au niveau du cou. Le bilan para clinique a objectivé une pancytopénie avec présence de nombreux éléments myéloïdes granuleux et érythrocytaire au myélogramme (Figure 2).

**Observation 2**: Il s'agit de l'enfant S M âgée de 06 ans issu d'un mariage consanguin de premier degré. L'examen clinique trouve un retard Staturo-pondéral (poids-2DS, taille-1DS), une dysmorphie faciale faite d'un visage triangulaire, hypertélorisme et micrognatisme et des taches café au lait au niveau du dos. Le bilan para clinique a objectivé une pancytopénie avec aplasie médullaire.

**Observation 3**: Il s'agit de l'enfant MS âgé de 07 ans, issu d'un mariage consanguin. L'examen clinique trouve un retard staturo-pondéral (poids-2DS, taille-2DS), une dysmorphie faciale faite de microcéphalie et visage triangulaire et une hyperpigmentation cutané au niveau du tronc. Le bilan para clinique a objectivé une pancytopénie et une hypoplasie médullaire.

**Figure 3:**
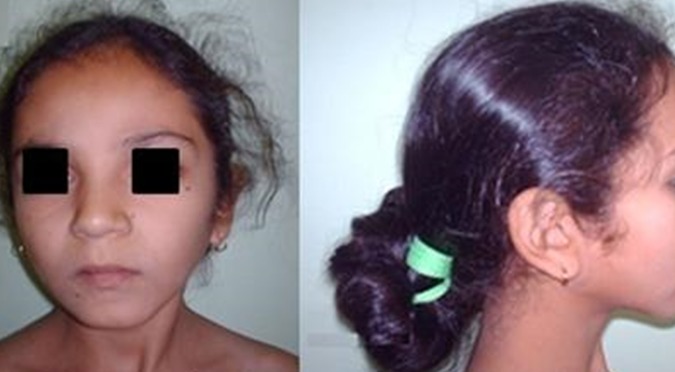
Dysmorphie, observation 4

**Observation 4**: il s'agit de l'enfant BY âgée de 4ans issu d'un mariage consanguin de premier degré consultant pour pneumopathies à répétition. L'examen clinique trouve un retard staturo-pondéral (poids-1DS, Taille-2DS) et une dysmorphie faciale faite de visage triangulaire avec traits fins. Le bilan para clinique a objectivé une pancytopénie et une moelle hemodiluée avec un excès relatif de lymphocytes et absence quasi-totale de mégacaryocytes au myélogramme (Figure 3).

**Figure 4:**
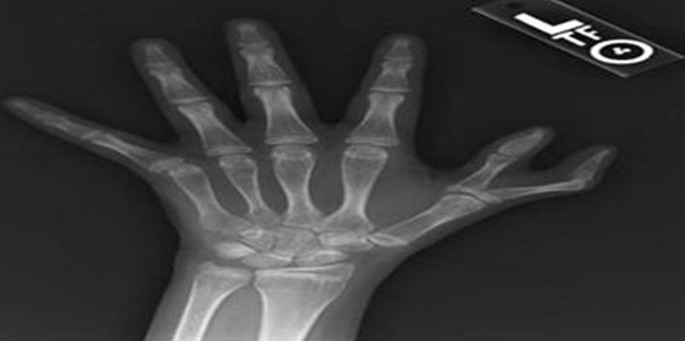
Pouce bifide, observation 5

**Observation 5**: Il s'agit de l'enfant DC âgé de 11, issu d'un mariage consanguin, consultant pour syndrome anémique. L'examen clinique trouve un retard staturo'pondéral (poids-3DS, taille-2DS), une dysmorphie faciale, pouce bifide bilatérale et taches café au lait au niveau de l'avant-bras droit. Le bilan para clinique a objectivé une pancytopénie avec aplasie médullaire (Figure 4).

**Observation 6**: Il s'agit de l'enfant ES âgé de 3 ans issu du mariage non consanguin. L'examen clinique trouve un retard staturo-pondéral (poids-3DS, taille-1DS), une dysmorphie faciale faite de microcéphalie et visage triangulaire et une cryptorchidie. Le bilan para clinique a objectivé une pancytopénie.

**Méthodes utilisées**: Devant ces tableaux cliniques et les résultats de l'hémogramme, du myélogramme et de la biopsie médullaire nous avons suspecté une anémie de fanconi. On a ainsi réalisé un caryotype sur des lymphocytes sanguins après culture pendant 72 heures en présence de Mitomycine C. Les cellules sont bloquées en métaphase par la colchicine. Après un choc hypotonique au KCI, les mitoses sont fixées par un mélange méthanol/acide acétique. Les préparations chromosomiques ainsi obtenues sont analysés de façon semi-automatique grâce à un logiciel spécialisé couplé à une caméra.

## Résultats

**Figure 5:**
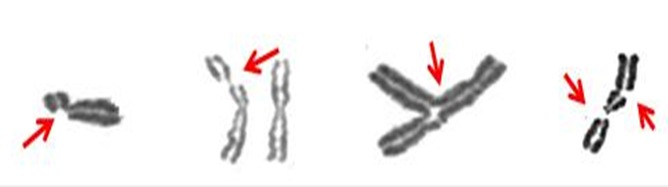
Différents aspects cytogénétiques d'une instabilité chromosomique après culture sous mitomycine C

L'analyse cytogénétique à la recherche d'instabilité chromosomique chez nos 6 patients a mis en évidence la présence d'une grande instabilité chromosomique après culture sous *Mitomycine C* en comparaison avec un témoin normal du même sexe et du même âge (Tableau 1). Ces résultats sont en faveur d'une anémie de Fanconi (Figure 5).

**Tableau 1:** Résultat obtenu après culture sous mitomycine C

Analyse cytogénétique	Cas 1	Cas 2	Cas 3	Cas 4	Cas 5	Cas 6
Caryotype métaphasique (Bande R)	46, XY	46, XX	46, XY	46, XX	46, XX	46, XY
Nombre de mitoses	60	55	59	40	50	50
Nombre de cassures	23	22	16	33	27	46
Nombre d’images radiales	2	3	0	0	0	0
Résultats	Grande instabilité chromosomique après culture sous *Mitomycine C* en comparaison avec un témoin normal					

## Discussion

L'anémie de Fanconi est une pathologie récessive rare (1/360.000 naissances) décrite en 1927 par Le pédiatre suisse Guido Fanconi. Elle est caractérisée par une pancytopénie progressive, l'association de diverses anomalies congénitales et une prédisposition au développement de pathologies malignes. Elle représente la forme la plus fréquente d'anémie aplasique congénitale. Son incidence est plus élevée dans deux groupes ethniques: chez les juifs ashkénazes et chez les Afrikaners, deux populations caractérisées par un taux important de consanguinité [7]. Nous avons constaté un pourcentage assez important de 83.3% soit 5 sur 6 cas de consanguinité dans notre série. L'âge moyen au moment du diagnostic est de huit ans. La découverte de la maladie après 20 ans est rare, Une trentaine de cas sont rapportés dans Ia littérature [8]. Une prédominance masculine est souvent rapportée. L'âge moyen de survie est de 25 ans. Dans notre étude on a trouvé une répartition égale du sexe et l'âge moyen est de 7,2. Il existe une très grande hétérogénéité des caractéristiques cliniques parmi les patients atteints d'anémie de Fanconi, même au sein d'une même famille. Les anomalies morphométriques (petit poids de naissance, retard de croissance, microcéphalie, microphtalmie, microstomie) ainsi que la dysmorphie faciale (visage fin, triangulaire, miniaturié) se retrouvent chez 60% des patients. Les anomalies de la pigmentation cutanée (60 à 80% des patients), souvent absentes à la naissance et durant la petite enfance, sont constituées de taches « café au lait » qui apparaissent sur un fond d'hyperpigmentation généralisée. Les anomalies squelettiques (50% des patients) sont le plus souvent limitées aux membres supérieurs où elles affectent typiquement le rayon radial notamment les anomalies du pouce (qui peut être absent, hypoplasique ou surnuméraire) [9-11]. Des anomalies rénales (aplasie, hypoplasie rénale, rein ectopique etc.) affectent un quart des patients, ils doivent être systématiquement recherchés par UIV. En dehors de ces quatre types d'anomalies, la plupart des autres systèmes peuvent être atteints, en particulier, le système gastro-intestinal, le système cardiopulmonaire et le système nerveux central [10].

Cependant une étude a montré que un tiers des patients atteints d'anémie de Fanconi n'ont pas d'anomalies congénitales évidentes. Le retard staturo-pondéral et la dysmorphie faciale sont présents chez tous nos patients, Les troubles de la pigmentation cutanée ont été observés chez 50% des cas Pourcentage proche mais légèrement inférieur (60 à 75%) à celui de plusieurs séries. Notre série a enregistré 1 cas de malformation osseuse à type de pouce bifide. La cryptorchidie, observée chez un patient, est rarement rapportée dans la littérature. Par contre les malformations digestives, qui touchent 14 à 20% des patients selon la littérature, n'ont pas été présentes dans notre étude, de même que les malformations cardiaques. Il faut souligner que si le phénotype clinique de l'AF est très variable, deux caractéristiques sériques sont présentes chez la quasi-totalité des patients: un titre élevé d'α-f'toprotéine, et un titre très élevé de TNF-α, cytokine pro-inflammatoire et cytotoxique. La plupart des patients atteints d'anémie de Fanconi développent des anomalies hématologiques. Les premiers signes de pancytopénie apparaissent le plus souvent entre trois et dix ans. La thrombopénie précède généralement la leucopénie qui elle-même précède l'anémie. Dans la majorité des cas il existe des signes d'érythropoïèse de stress. Au stade d'aplasie, la moelle osseuse est hypocellulaire et graisseuse. Des anomalies cytogénétiques clonales peuvent être retrouvées chez les patients présentant ou non une insuffisance médullaire et augmentent le risque de développer un syndrome myélodysplasique ou une leucémie myéloïde aiguë. Sur le plan biologique, dans notre série conformément à la littérature, la lignée plaquettaire était la plus touchée (83,3% des cas). La neutropénie était également constante (66.7% des cas). L'anémie était présente dans 83.3% des cas, elle était toujours normo-chrome et le plus souvent macrocytaire. La biopsie médullaire et myélogramme réalisés dans notre série montrent une moelle normale ou hypoplasique. l'AF est aussi génétiquement hétérogène. L'existence d'au moins deux groupes de complémentation a été reportée en 1985. Actuellement on considère qu'il y a au moins 15 groupes de complémentation différents [7].

Le groupe FA-A est le plus commun, représentant environ 65% des individus. Les groupes FA-C et FA-G représentent chacun environ 15% des patients, les individus appartenant aux autres groupes sont par conséquent relativement rares. Le modèle de transmission est de type mendélien récessif pour tous les groupes de complémentation à l'exception du groupe FA-B, dont la transmission est liée au sexe. Les individus hétérozygotes (fréquence estimée à 1/300) sont asymptomatiques et aucune approche, à part la recherche systématique de mutations dans les gènes FANC à l'intérieur de familles touchées par le syndrome, ne permet leur dépistage [5, 6]. La variation interindividuelle peut aussi être observée dans le cas d'une même mutation touchant un même gène. L'inactivation d'un gène de l'AF s'accompagne d'une fertilité réduite à cause d'anomalies dans le développement des cellules germinales. Cependant, plusieurs grossesses ont été rapportées ainsi que de rares exemples de paternité [12]. Les protéines FANC font partie d'un réseau complexe auquel participent des composants de la chromatine (H2AX, BRG1), des protéines de signalisation (ATM, ATR), des protéines détectrices des dommages ou adaptatrices (RPA, le complexe RAD50-MRE11-NBS1, BRCA1), des hélicases (BLM, des protéines impliquées dans des voies de réparation de l'ADN comme le NER (XP-F) et la recombinaison homologue (RAD51), ou dans la tolérance des lésions (synthèse translésionnelle ou TLS). Toutes ces protéines sont impliquées dans la réponse aux dommages de l'ADN. Les protéines FANC agiraient en contrôlant et en coordonnant le bon déroulement du processus de réparation et d'assemblage de différents composants au niveau du site endommagé. Classiquement, le diagnostic repose sur l'analyse des aberrations chromosomiques induites par une classe d'agents endommageant l'ADN connue sous le nom d'agents pontants. En effet, après exposition à des drogues comme la mitomycine C, le diépoxybutane ou les moutardes azotées, la fréquence des aberrations chromosomiques induites dans les cellules AF est significativement supérieure à celle observée dans les cellules issues de donneurs normaux. De plus, les cellules AF traitées aux agents pontant l'ADN présentent des anomalies chromosomiques, des cassures chromatidiennes et chromosomiques (fragments, figures tri-et quadriradiales) très rarement observées dans les cellules normales.

Une autre approche diagnostique est la cytométrie de flux. Cette méthode permet de détecter les cellules accumulées en phase G2 du cycle cellulaire, elle peut porter sur des cellules non traitées ou exposées à des agents pontant l'ADN. En effet, la phase G2 du cycle cellulaire est significativement plus importante dans les cellules AF. Suite au clonage des gènes FANC, une analyse moléculaire ou biochimique peut se substituer ou s'ajouter à l'analyse cytogénétique. Le diagnostic anténatal peut se faire soit entre neuf et 12 semaines par biopsie des villosités chorioniques, soit vers 16 semaines par amniocentèse. L'AF est associée à un risque très élevé de développer un cancer, on estime qu'environ 30% des patients développeront une tumeur au cours de leur vie: une leucémie myéloïde aiguë (15-20% des patients), un cancer de la cavité buccale ou un hépatocarcinome (5% des patients). Le risque de leucémie chez les patients AF est estimé être 15 000 fois plus élevé que dans la population générale appartenant à la même classe d'âge [9]. Jusqu'à ce moment-là, aucun cas de notre série n'a présenté une complication associée à cette maladie. Des traitements avec des androgènes ou des cytokines permettent d'obtenir une amélioration transitoire de la formule sanguine, mais à terme deviennent inefficaces. L'utilisation d'une thérapie neutralisant le TNF-α, dont le niveau sérique chez les patients est très élevé et qui pourrait participer au développement de l'aplasie médullaire du syndrome, est actuellement à l'étude aux États-Unis et en Italie. La greffe médullaire est la seule possibilité de guérir l'aplasie médullaire des patients et de prévenir la leucémie. Selon l'EUFAR, le taux de survie à cinq ans en cas d'allogreffe de donneur apparenté HLA-identique est de 74%. L'alternative à la greffe médullaire pour la majorité des patients n'ayant pas de donneur compatible, est la thérapie génique. Toutefois comme la greffe médullaire, ce type de traitement ne permet pas de corriger les anomalies de développement ni de diminuer le risque de voir apparaître des tumeurs solides. La prise en charge extra-hématologique associe le dépistage des malformations et déficits d'organes à une surveillance de l'apparition de lésions précancéreuses ou tumorales. Dans notre série un seul patient a pu bénéficier de la greffe de moelle et deux cas de notre série ont été transfusés par culot globulaire; mais aucun d'eux n'a reçu un traitement par les androgènes (Androgénotherapie), corticothérapie ou thérapie génique. Le conseil génétique rejoint celui des maladies autosomiques récessives et vu l'hétérogénéité clinique de cette pathologie, la recherche d'instabilité chromosomique doit être instauré chez les membres de la famille du patient.

## Conclusion

L'anémie de Fanconi est le centre d'intenses recherches tant fondamentales que cliniques. Son traitement a nettement progressé, entre autre, par l'amélioration du régime de préparation à la greffe et des techniques de sélection des donneurs. La thérapie génique est une autre voie de traitement qui fait l'objet de plusieurs études. Le développement à long terme de carcinomes est une complication à laquelle ils seront de plus en plus souvent confrontés et pour lequel il n'existe pas actuellement de mesure préventive. Le but de cet article est la mise à jour de nos connaissances sur la génétique de l'Anémie de Fanconi et à travers ces six observations nous illustrons le rôle de la cytogénétique dans le diagnostic et le conseil génétique.

### Etat des connaissances actuelles sur le sujet

- L'anémie de Fanconi est une maladie récessive associée à une instabilité chromosomique;
- La cytogénétique confirme le diagnostic;
- Le conseil génétique rejoint celui des maladies autosomiques récessives.

### Contribution de notre étude à la connaissance

- Première série marocaine;
- La mise à jour de nos connaissances sur la génétique de l'Anémie de Fanconi;
- Illustrer le rôle de la cytogénétique dans le diagnostic et le conseil génétique.

## Conflits d’intérêts

Les auteurs ne déclarent aucun conflit d'intérêts.
